# Coronary spastic angina after the administration of intravenous immunoglobulin in myasthenia gravis: a case report

**DOI:** 10.1186/s12883-020-01901-2

**Published:** 2020-08-28

**Authors:** Masaru Yanagihashi, Ryuichi Okamoto, Harumi Morioka, Masahiro Sawada, Shingo Matsumoto, Takanori Ikeda, Osamu Kano

**Affiliations:** 1 Division of Neurology, Toho University Omori Faculty of Medicine, 6-11-1, Omorinishi, Ota-ku, Tokyo, 143-8541 Japan; 2 Division of Cardiovascular Medicine, Toho University Omori Faculty of Medicine, Tokyo, Japan

**Keywords:** Coronary spastic angina (CSA), Intravenous immunoglobulin (IVIG), Myasthenia gravis (MG)

## Abstract

**Background:**

Myasthenia gravis (MG) is an autoimmune disease caused by antibodies that block or destroy nicotinic acetylcholine receptors at the neuromuscular junction. Most of MG patients need immunosuppression agents in addition to treatments that alleviate the symptoms. Intravenous immunoglobulin (IVIg) and plasma exchange are specific treatments given to patients with severe MG and myasthenia gravis crisis. IVIg therapy can cause an increase in serum viscosity; therefore, the risk for thromboembolic events, such as stroke, myocardial infarction, and pulmonary embolism, are reported after IVIg therapy.

**Case presentation:**

An MG patient was treated with pyridostigmine bromide and prednisolone. The patient’s symptoms worsened 26 days after the commencement of treatment and was presented with head drop and dyspnea. The patient was diagnosed with MG crisis and IVIg was initiated. However, the patient reported chest pain and dyspnea 3 days after IVIg had started. An electrocardiogram (ECG) revealed ST elevations in leads II, III, and aVF. A cardiac catheterization was performed and stenosis, obstruction, and sclerosis were ruled out. Glyceryl trinitrate relieved the patient’s symptoms, suggesting coronary spastic angina (CSA).

**Conclusions:**

We report the first case of CSA after IVIg. Practitioners should be aware of the potential risks of CSA when administering IVIg for MG patients, in particular in old patients with vascular risk factors.

## Background

Myasthenia gravis (MG) is an autoimmune disorder of the neuromuscular junction (NMJ), manifesting as skeletal muscle fatigable weakness. In most cases of MG, the number of available acetylcholine receptors is decreased due to the autoimmune attack. However, the muscle-specific kinase, lipoprotein receptor-related protein 4, agrin, titin, and ryanodine receptors are targeted in some cases of MG [1, 2].

Acetylcholinesterase inhibitors and immunosuppressive agents such as prednisolone and azathioprine are effective in treating MG. However, patients with MG crisis that require intubation or noninvasive ventilation should receive immunosuppressive agents and intensive care [2]. In MG exacerbation and crisis, intravenous immunoglobulin (IVIg) and plasma exchange are recognized as effective therapies, providing benefits within 2 weeks of treatment commencement [3, 4]. IVIg is often regarded as convenient and safe for most MG crisis cases, but severe side effects including anaphylactic reactions, aseptic meningitis, acute renal failure, and thromboembolic events such as stroke, myocardial infarction, deep vein thrombosis, and pulmonary embolism have been reported [5].

We present the first reported case of coronary spastic angina (CSA) following IVIg administration to an MG crisis patient who suffered from head drop and dyspnea.

## Case presentation

An 87-year-old woman with osteoporosis and a lumbar compression fracture was transferred to our hospital for suspected MG due to a gradual increase of limb weakness and an anti-acetylcholine receptor (AChR) antibody level. The patient underwent surgery for a pressure ulcer on her lumbar spine 8 months prior. Her medical history was significant for falx meningioma, right kidney tumor, colorectal polyp removal, bile cyst enucleation, and diabetes mellitus.

The patient had noticed grip weakness for more than a year, and bilateral ptosis and diplopia for the past 3 months. Swallowing was normal, but she had difficulty chewing. The patient was a non-smoker, but her pulmonary capacity was decreased due to a restrictive ventilatory disorder. The patient’s AChR antibody increased to 75 nmol/L. Repetitive nerve stimulation electromyogram was performed on right accessory nerve, facial nerve and ulnar nerve. Waning in the orbicularis muscle (24% decrement) and abductor pollicis (19% decrement) was observed on 3 Hz stimulation. Computed tomography of the chest revealed no thymoma. The patient was diagnosed with MG, and her quantitative myasthenia gravis (QMG) score was 18 points. She was prescribed with an acetylcholinesterase inhibitor, pyridostigmine bromide (120 mg per day), and increments of prednisolone, starting at 5 mg every other day and titrated up to 10 mg once a day for 2 days. However, after 26 days of therapy, the patient’s symptoms worsened, with head drop and gradually fluctuating dyspnea; thus, the patient was diagnosed with MG crisis and we initiated IVIg (400 mg/kg per day), continuous infused at 500 mL per day.

**Fig. 1:**
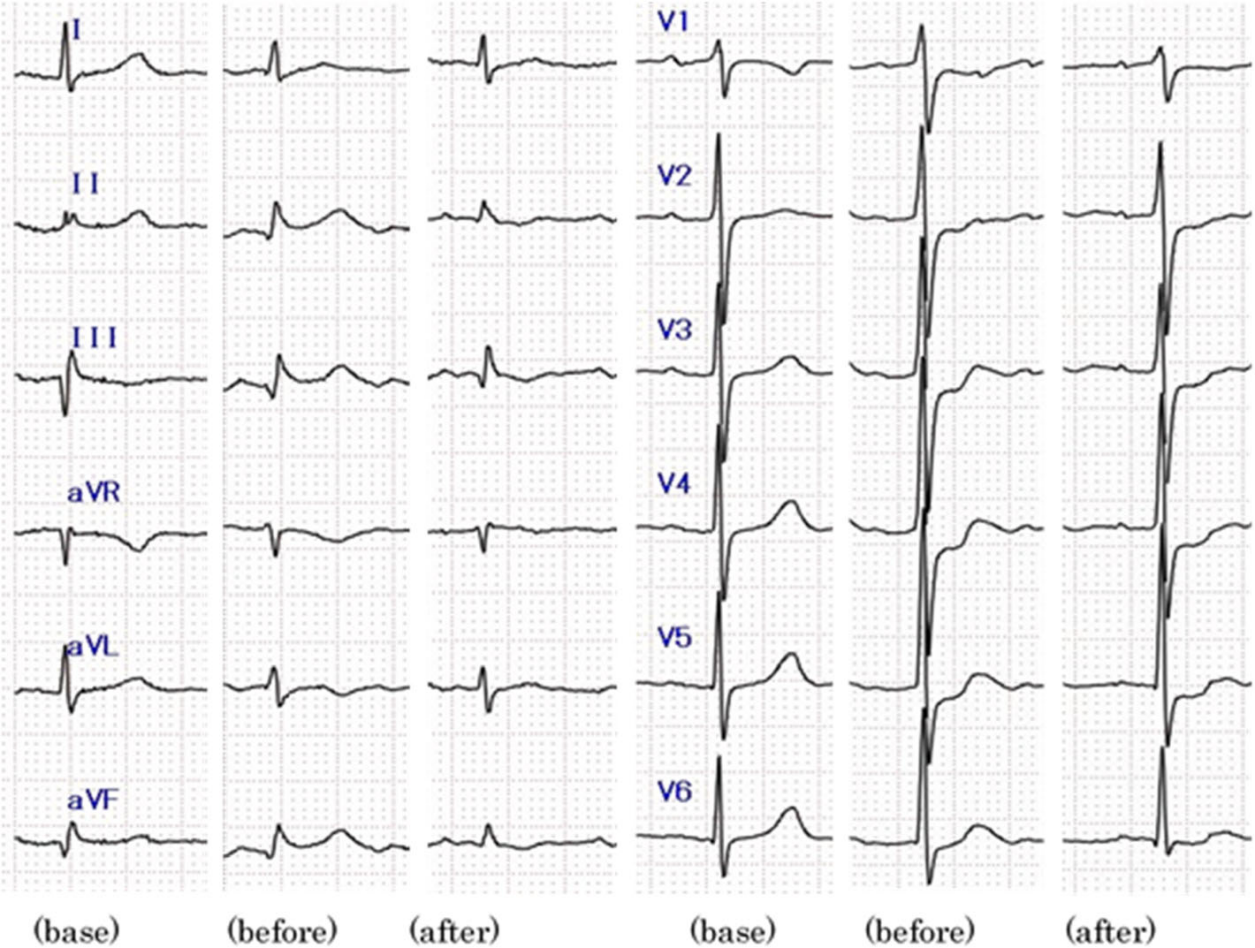
Electrocardiograms recorded at baseline (base) and at angina pectoris. ST elevation is seen in leads II, III, and aVF (before), and improvements in ST elevations are seen after administration of glyceryl trinitrate (after)

**Fig. 2:**
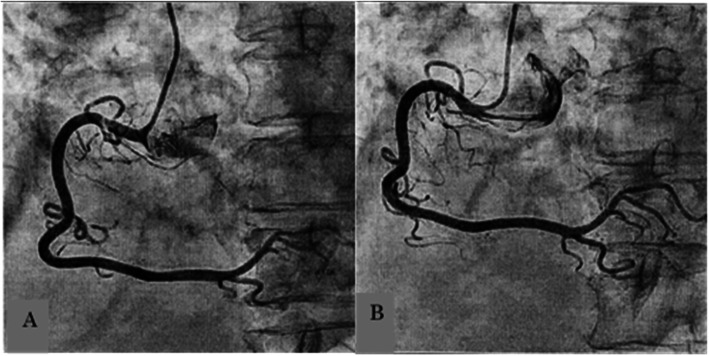
Cardiac catheterization shows the right coronary artery in spasm before treatment (**a**) and relieved after administering glyceryl trinitrate (**b**)

Three days after starting IVIg, the patient reported sudden chest pain and dyspnea in the evening. She had taken her last dose of pyridostigmine bromide after lunch that day. An electrocardiogram (ECG) revealed ST elevations in leads II, III, and aVF (Fig. 1). A cardiac catheterization was performed and stenosis, obstruction, and sclerosis were ruled out. Glyceryl trinitrate (48 mg per day), improved the patient’s symptoms, which suggested that the coronary arteries were in spasm (Figs. 1, and 2). The patient was started on nicorandil and has had no recurrence of chest pain for 1 year.

## Discussion and conclusion

MG treatment includes oral acetylcholinesterase inhibitors such as pyridostigmine bromide and immunosuppressive drugs. Moreover, patients with MG exacerbation or crisis are treated with intubation or noninvasive ventilation, IVIg, and plasma exchange. We decided to administer IVIg in this case when the patient exhibited head drop and dyspnea as signs of MG crisis. Previously, stroke, myocardial infarction, and other thrombotic complications have been reported as rare but severe side effects of IVIg, due to the associated increase in blood viscosity [5, 6]. In this case, we considered the patient’s chest pain and dyspnea to be acute ischemic heart disease or pulmonary embolism in response to IVIg. We could not perform enhanced computed tomography of the chest and abdomen to exclude pulmonary embolism, pneumothorax and aortic dissection. However, the improvement seen with the injection of glyceryl trinitrate would suggest coronary artery spasm rather than stenosis or obstruction.

Previous reports have suggested that there is a deficiency of nitric oxide (NO) activity in the endothelial cells of the coronary arteries with CSA [7]. Moreover, the spastic arteries are sensitive to nitroglycerin, a vasodilator, and to the effect of acetylcholine, a parasympathetic neurotransmitter, which can induce vasospasm by constricting the vascular smooth muscle when the endothelium is damaged [7–11]. Other reports showed that atherosclerotic lesions detected by intravascular ultrasound may be sites of focal vasospasm, even in the absence of significant angiographically confirmed coronary disease [12]. The patient had diabetes mellitus, so there may have been undetected atherosclerotic lesions at the site of the spasm.

A case of CSA induced by acetylcholinesterase inhibitor (ambenonium chloride) had been reported in an MG patient 3 weeks after dosing [13]. The patient in this report had taken pyridostigmine bromide, an acetylcholinesterase inhibitor, for 25 days, with her last dose several hours before CSA symptoms were noted. IVIg therapy in severe MG and MG crisis shows rapid effect, so there is no speculation, we hypothesized that the CSA might be induced by IVIg rather than the acetylcholinesterase inhibitor.

As shown in previous studies, IVIg has multiple modes of action, which include activation of bacterial phagocytosis, Fc receptors blockade, complement downregulation, suppression of cytokine activity modulation of dendritic cells, and T and B cell activation and differentiation [14]. In addition, IVIg may induce the production of vasoconstrictive cytokines, arterial vasospasm, and disrupt atherosclerotic plaques leading to thrombotic events [15, 16]. IVIg therapy causes an increase in serum viscosity; therefore, the risk for thromboembolic events such as myocardial infarction, pulmonary embolism, and stroke, especially in patients with risk factors for cerebral vascular disease as in our case, must be considered. Further study is required to confirm the relationship between IVIg and CSA. Practitioners should be aware of the potential risk of CSA, and we recommend caution when administering IVIg to treat MG patients, particularly in elderly patients with vascular risk factors and several comorbidities.

## Data Availability

All data resides within the author’s premises and is available to the author upon reasonable request.
